# Routing and Scheduling Algorithms for WirelessHART Networks: A Survey

**DOI:** 10.3390/s150509703

**Published:** 2015-04-24

**Authors:** Marcelo Nobre, Ivanovitch Silva, Luiz Affonso Guedes

**Affiliations:** 1 Department of Computer Engineering and Automation, Federal University of Rio Grande do Norte, Campus Universitario, 59078-900 Natal, Brazil; E-Mail: affonso@dca.ufrn.br; 2 Metropole Digital Institute, Federal University of Rio Grande do Norte, Campus Universitario, 59078-900 Natal, Brazil; E-Mail: ivan@imd.ufrn.br

**Keywords:** industrial wireless sensors networks, WirelessHART, routing, scheduling, mesh networks

## Abstract

Wireless communication is a trend nowadays for the industrial environment. A number of different technologies have emerged as solutions satisfying strict industrial requirements (e.g., WirelessHART, ISA100.11a, WIA-PA). As the industrial environment presents a vast range of applications, adopting an adequate solution for each case is vital to obtain good performance of the system. In this context, the routing and scheduling schemes associated with these technologies have a direct impact on important features, like latency and energy consumption. This situation has led to the development of a vast number of routing and scheduling schemes. In the present paper, we focus on the WirelessHART technology, emphasizing its most important routing and scheduling aspects in order to guide both end users and the developers of new algorithms. Furthermore, we provide a detailed literature review of the newest routing and scheduling techniques for WirelessHART, discussing each of their features. These routing algorithms have been evaluated in terms of their objectives, metrics, the usage of the WirelessHART structures and validation method. In addition, the scheduling algorithms were also evaluated by metrics, validation, objectives and, in addition, by multiple superframe support, as well as by the redundancy method used. Moreover, this paper briefly presents some insights into the main WirelessHART simulation modules available, in order to provide viable test platforms for the routing and scheduling algorithms. Finally, some open issues in WirelessHART routing and scheduling algorithms are discussed.

## 1. Introduction

Wireless networks are a current trend, with varied applications, including biomedicine, residential automation, environmental surveillance and video security monitoring [1]. Consequently, industrial environments are also benefiting from such technology. The history of industrial wireless technologies (IWT) recalls the fieldbus war (1986–2002) [2], when several technologies appeared as *de facto* standards for industrial networks (Modbus [3], Profibus [4], Fieldbus Foundation [5], HART [6], to name just a few). All of these technologies were based on structured cabling, and they had as a goal creating a universal protocol to meet all types of industrial demands [7]. Recently, industrial wireless technologies have attracted great interest, providing an environment with fewer cables, lowering the installation/maintenance costs and providing greater flexibility to the network [8]. However, despite the appearance of wireless technologies, the scenario has not been very different. A variety of wireless technologies coexist, and others are yet to be seen. There are even examples where wireless technology is used as an extension for a traditional wired technology, as reported by [9]. Establishing a conservative scenario, just one wireless solution will hardly be the universal standard for the industry. It is wiser to think that several solutions will coexist, each one having its own advantages and disadvantages and its specific niche applications.

The literature contains many comparisons between the wireless technologies. A comparison of ISA100.11a (International Society of Automation 100) with WirelessHART can be seen in [10–12]. The Chinese standard WIA-PA (Wireless Networks for Industrial Automation-Process Automation) is added to the comparison with WirelessHART and ISA100.11a in [13]. In [14], a user perspective for the use of wireless technology in a power plant scenario is discussed, in order to aid the decision making process when choosing a wireless technology for this kind of environment. Furthermore, [15] presents a successful test where WirelessHART is used as a substitute for Profibus in a closed control loop. Among the aforementioned technologies, WirelessHART is pointed out by [16] as a feasible solution for wireless industrial networks, which will be the focus of the present paper.

Industrial wireless sensor networks (IWSN) are commonly planned topologies, having low mobility nodes, and some devices in the network may be powered by batteries. However, the different applications and situations may require the optimization of specific characteristics, such as transmission delay [17,18], energy consumption [19] and data reliability [20–23]. The routing and scheduling algorithms used by the network are very important: they determine the routes, channels and the redundancy policy of the network, thus directly affecting the aforementioned network characteristics. Thus, there is a need to adequately combine WirelessHART technology with the proper routing and scheduling algorithms in order to enhance the specific characteristics that each application needs. The present paper aims to analyze the most recent approaches to routing and scheduling issues for WirelessHART and to provide a clear view of the actual scenario, as an aid for researchers and industries on what routing and scheduling schemes would fit their needs.

Several surveys have been made for routing in wireless sensor networks (WSN). The surveys presented in [24–26] do not focus on specific characteristics, providing a broader overview of the present state-of-the-art. As the field of routing schemes is a wide area, there are surveys that deal specifically with protocols that present clearer optimization objectives. The surveys in [27,28] focus on energy-efficient protocols, especially the latter one, which classifies more than fifty routing schemes. The [29] survey focuses on fault-tolerant protocols, evaluating issues, such as energy consumption, memory use, recovery time and overhead. The survey presented by [30] focuses on the routing protocols developed for wireless body sensor networks (WBSNs), a subset of the WSNs responsible for monitoring the vital signs of patients and accordingly routing these data towards a sink. According to [30,31], routing algorithms for WBSNs need to address some specific issues, such as: frequent topology partition due to body postural movements; devices with very limited resources in order to be attached to a human body; new nodes must be able to enter the network quickly and easily; integrity of the data and patient privacy issues are critical; and the networks are often linked to external healthcare systems through the Internet. Those issues are not as important or even do not exist in IWSN, thus those algorithms cannot viably be implemented in a WirelessHART system without further adaptation and, thus, are not suitable for the scope of the present paper. The work in [32] presents a survey in order to provide insight into routing protocols designed specifically for large-scale generic WSNs. As examples for large-scale WSN applications, we have intrusion detection, weather monitoring and disaster monitoring. Finally, the survey presented by [33] has the objective of presenting the state-of-the-art of duty-cycled wireless sensor networks. The author defines duty-cycled networks as those in which the nodes turn off their radios when no sensing or communication tasks are required and go to sleep, to wake up later in order to sample the environment and check for any channel activity.

For scheduling, the literature has fewer surveys than for routing. The work in [34] describes the main WSN properties that are important for the design of a time division multiple access (TDMA) protocol and also outlines the main protocols available for WSN. The comparison of the protocols is based on latency, channel interference, synchronization time needed, communication patterns supported by the algorithm and the adaptability to changes in the topology. A survey of bandwidth resource allocation and scheduling is presented by [35], addressing heuristics for slot time allocation on multiple hops. Finally, [36] presents a survey that analyzes algorithms to support the allocation of a guaranteed time slot (GTS; see [37]) to the devices during the contention-free period part of the superframe [38] in IEEE 802.15.4-based networks.

Although this is a very rich environment, none of the aforementioned surveys are technology focused, providing an overview for generic WSNs. In order to attend to users who have already chosen the WirelessHART technology, we will provide an analysis and comparison of the newest routing and scheduling protocols for this technology.

The remainder of this paper is organized as follows. In Section 2, we present a brief overview of the WirelessHART technology. Sections 2.1 and 2.2 emphasize the TDMA technique, used in the MAC layer, and the IEEE 802.15.4 physical layer structures, common to the other current IWSN technologies (ISA100.11a [39] WIA-PA [40]). As the main focus of the present paper, the scheduling and routing of the WirelessHART are respectively detailed in Sections 2.2.1 and 2.2.2. Section 3 presents a comparative study of the state-of-the-art of routing algorithms. A similar description and comparison is presented in Section 4 for the state-of-the-art WirelessHART scheduling algorithms. In Section 5, we will present the main WirelessHART simulators available for algorithm validation purposes. Finally, some concluding remarks are provided in Section 6.

## 2. WirelessHART

As the WirelessHART technology is the basis for the present paper, in this section, we will present a brief history of the technology and how it is organized. Furthermore, we will present the TDMA technique and the IEEE 802.15.4 physical layer, since those are common traits among WirelessHART and the main IWSN nowadays (WIA-PA and ISA100.11a). Then, we will proceed to the main focus of the present paper, by emphasizing the specific traits of WirelessHART routing and scheduling.

**Figure 1:**
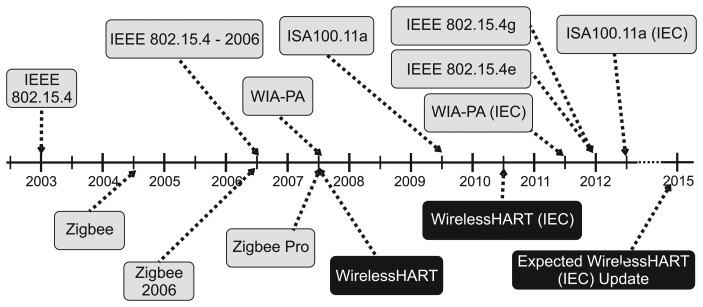
Historical development of the industrial WSN (IWSN) technologies.

WirelessHART is an extension of the HART protocol to support wireless communication. In September, 2008, the WirelessHART specification (HART 7.1) was approved by the International Electrotechnical Commission (IEC) as a publicly available specification (IEC 62591) [6]. In April 2010, WirelessHART was the first industrial wireless communication technology to attain this level of international recognition, as can be seen in Figure 1 [16]. A WirelessHART IEC update is expected for 2015.

**Figure 2:**
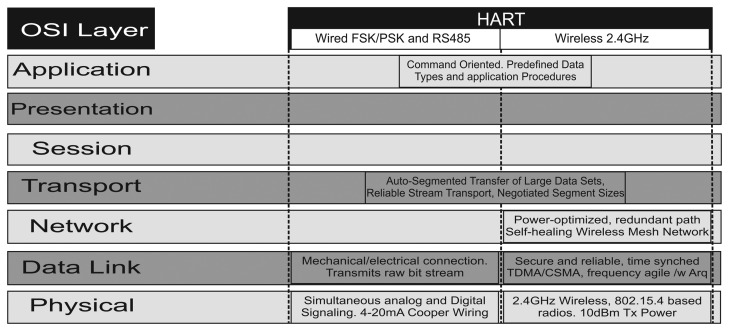
WirelessHART architecture compared to wired HART and the OSI stack. OSI, Open Systems Interconnection.

WirelessHART technology implements five layers: a physical layer, a data link layer, a network layer, a transport layer and an application layer. Thus, when compared to the traditional OSI (Open Systems Interconnection) stack model, the presentation layer and session layer do not have direct equivalents in the WirelessHART stack [41], as can be seen in Figure 2. Figure 2 also presents the equivalences and differences of the traditional wired HART technology and the WirelessHART. It is worth noting that WirelessHART devices are fully compatible with a traditional HART network [7].

**Figure 3:**
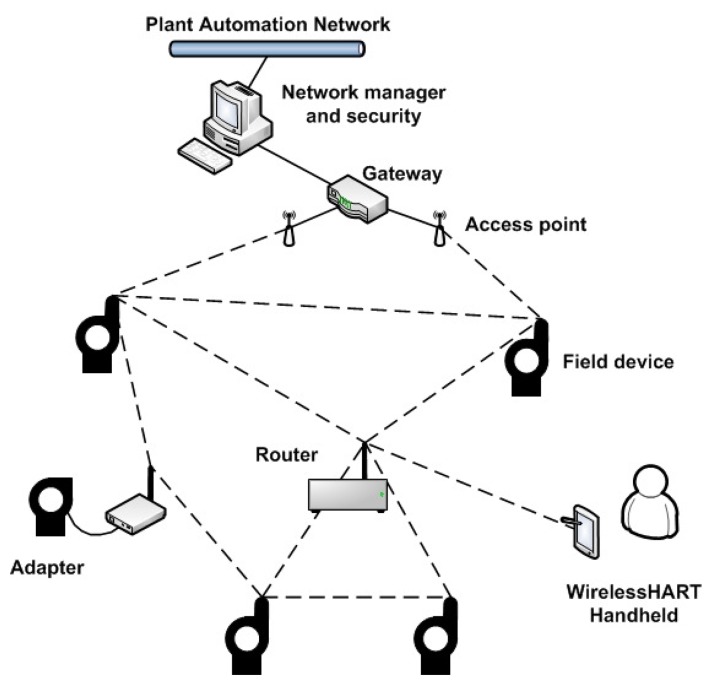
WirelessHART devices.

WirelessHART defines eight types of devices: network managers, network security devices, gateways, access points, field devices, adapters, routers and handheld devices. All devices are connected to the wireless network to build a mesh-type topology (see Figure 3) and implement basic mechanisms to support network formation, maintenance, routing, security and reliability. The communications are organized by the network manager, which centralizes the information from all of the network devices (including joining devices) to make the routing and scheduling function.

Although WirelessHART has a physical layer based on IEEE 802.15.4, it implements its own medium access control (MAC) sublayer. The MAC layer uses the frame structure described in Section 2.1, but naming it a superframe. Superframes are composed of slots; the number of slots indicates the periodicity of the superframe. In order to support different transmission intervals, a WirelessHART network can use multiple superframes with different numbers of slots. Each slot has a fixed duration of 10 ms, which is enough time to transmit a packet and receive an acknowledgment message (the maximum packet size is 133 bytes, including headers). A slot supports up to 15 channels. Thus, theoretically, 15 devices can simultaneously transmit in the same slot time. The standard uses a mechanism of frequency hopping and a blacklist channel to minimize the influence of noise in network operation and consequently increase the reliability of the communication. As the protocol is TDMA-based, the source and destination stations have specific times to transmit and sense the medium. More details about the values of those times can be found in [6].

### 2.1. Time Division Multiple Access

In a TDMA system, each device uses the whole channel bandwidth for a fraction of time [42]. Those fractions of time are called slots; a slot is the period of time in which the process data are transmitted. In each slot, only one station transmits to one (unicast) or more destinations (multicast or broadcast), in order to avoid collisions.

**Figure 4:**
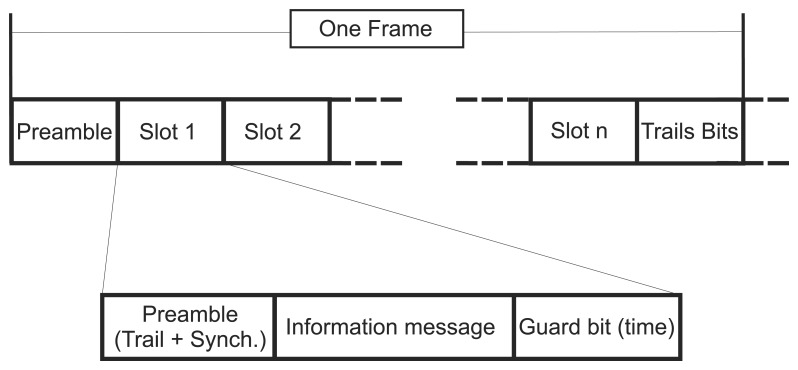
Time division multiple access (TDMA) frame organization.

Several slots make up a frame (which may also be called a superframe). The frame is represented in Figure 4. The synchronization and address information are carried in the preamble and the guard times at the end of the slots, to avoid crosstalk among the transmissions.

The TDMA is attractive for industrial communications, since it is a deterministic system once each device has its specific determined slot time in which to transmit. Furthermore, TDMA offers the opportunity for frame-by-frame monitoring of the bit error rate and signal strength, once the system has information about which device and when the device should communicate [42].

### 2.2. The IEEE 802.15.4 Physical Layer

**Figure 5:**
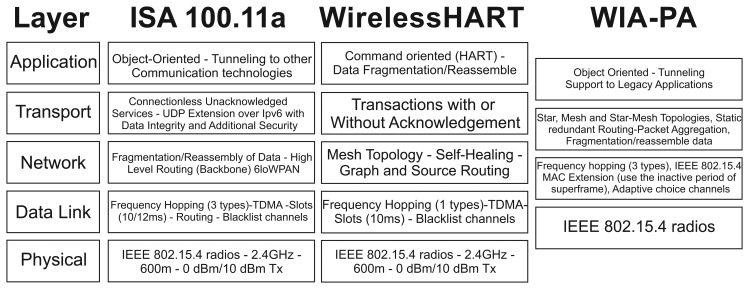
A layer-to-layer comparison of WirelessHART, ISA100.11a and WIA-PA [7,44].

As a common aspect for the aforementioned main industrial technologies, all of them adopt the IEEE 802.15.4 physical layer [43] (see Figure 5) and implement upper layers of their own. The low rate wireless personal area networks (LR-WPANs) were designed to support devices with limited physical features (low energy consumption, a communication range of a few meters, low power processing and reduced memory), enabling the development of pervasive applications in diverse areas (forest fire and landslide detection, greenhouse and industrial monitoring, passive localization and tracking, *etc.*). When an LR-WPAN is designed for an industrial environment, it is called an industrial wireless sensor network.

The standard for LR-WPANs is IEEE 802.15.4, which specifies the physical and data link layers. This standard was initially approved in 2003; however, its more recent version was approved in 2011 [6]. It is considered a *de facto* standard for wireless sensor networks.

The IEEE 802.15.4 physical layer can operate in three distinct frequency bands: the 2450-MHz ISM band (worldwide) with 16 channels, the 915-MHz band (in the U.S. only) with 10 channels and the 868-MHz band with only one channel (European and Japanese standards). All of them use the direct sequence spread spectrum (DSSS) access mode. Signaling in the 2450- MHz band is based on Orthogonal Quadrature Phase Shift Keying (O-QPSK), while the 868-/915-MHz bands rely on Binary Phase Shift Keying (BPSK). The throughputs of the bands are 250 kbps, 40 kbps and 20 kbps, respectively. Besides the management of the physical layer, services include the activation and deactivation of the radio transceiver, energy detection (ED) within the current channel, link quality indication (LQI) for received packets, clear channel assessment (CCA) for carrier sense multiple access with collision avoidance (CSMA-CA), channel frequency selection and data transmission and reception. The main features present in the IEEE 802.15.4 physical layer are summarized in Table 1.

**Figure 6:**
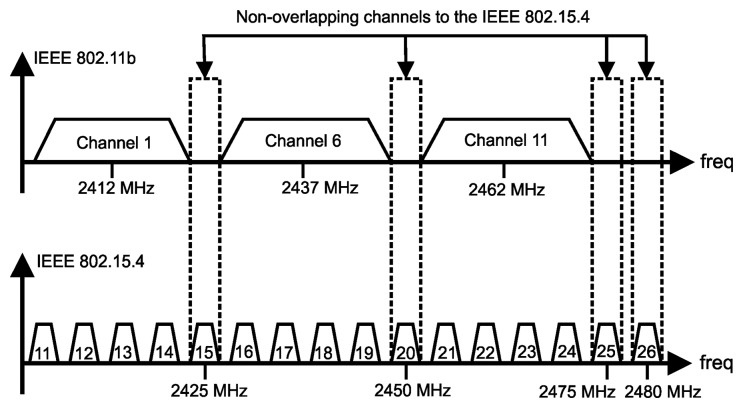
Channel overlap for 802.15.4 and 802.11b.

Although the physical layer can support three frequency bands, the 2450 MHz ISM band is by far the most frequently used. The use of this band generates a coexistence problem in the sense that other wireless technologies also employ the same frequency band (e.g., IEEE 802.11 and Bluetooth). In relation to coexistence with the IEEE 802.11 devices, it is possible to carry out a configuration, so that there are four non-overlapping IEEE 802.15.4 channels, as described in Figure 6. Obviously, as only four channels are available in this scenario, it is expected that the transmissions have a higher probability of suffering interference than a scenario where 16 channels are available [45]. On the other hand, a minor impact is expected when coexistence with Bluetooth devices is considered, because in the Bluetooth standard, the frequency band is modified 1600 times per second. Thus, it is more likely that the interferences occur in the first transmission attempts. However, an efficient retransmission mechanism can be implemented in the IEEE 802.15.4 to mitigate this interference [46].

#### 2.2.1. WirelessHART Scheduling

For the scheduling process, the WirelessHART specification [6] only suggests an algorithm that meets the requirements defined by the specification. The requirements are presented in Table 2, and those requirements must be implemented by the algorithms that intend to work with WirelessHART.

**Figure 7:**
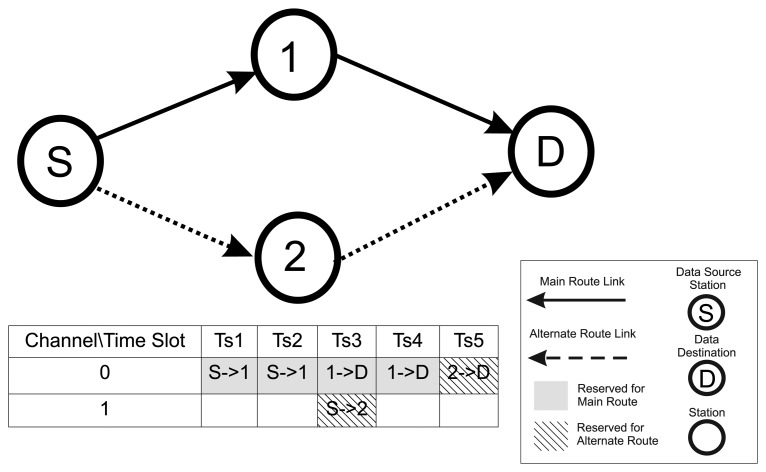
An example of scheduling on a simple topology.

This algorithm suggestion states that an alternate route should be used, if possible, beyond the main route from the data source to the data destination. For each packet to be transmitted by a station, two time slots must be reserved for scheduling purposes: the first for the transmission and a later one for a possible retry (both through the main route). If an alternate route is defined for that link, a third copy packet must be sent through that alternate route. For the sake of understanding the scheduling approach, a simple example is presented in Figure 7.

In the first time slot of the example described in Figure 7, the data are produced at the source station “S” and sent to the destination station “D” using the main route S-1-D and the alternate route S-2-D. According to the aforementioned scheduling algorithm, the two first time slots (Ts1 and Ts2) are reserved for the packet's transmission and retransmission through the main route on Channel 0. On the other hand, Ts3 reserves Channel 1 for the transmission through the alternate route. Again, Channel 0 in Ts3 and Ts4 is reserved for the main route's transmission and retransmission. Finally, in Ts5, Channel 0 is reserved for the transmission of the alternate route's sole try.

It is worth noting that after the definition of the channel offset, WirelessHART uses time slotted channel hopping (TSCH) [47] to determine the actual channel in which the data will be transferred in that slot. The equation that determines the actual transmission channel can be seen in Equation (1) and is based on the absolute slot number (ASN), which identifies one slot time, the channel offset defined in the scheduling and the number of actual available channels without the blacklisted ones (NumberOfChannels).

(1) ActualChannel=(ANS+ChannelOffset)%NumberOfChannels

#### 2.2.2. WirelessHART Routing

The scheduling must be built over defined routes. WirelessHART possesses two main ways to route packets: graph routing and source routing. Graph in the WirelessHART context means a set of edges that connect the devices on the network. In graph routing, the edges are created by the network manager and downloaded to each device. The sender device writes a graph ID into the header of the message to send a packet. As the packets arrive at a station, that station forwards (or consumes, if the station is the final destination) the packet according to the previously stored data. Each intermediate device can be configured with multiple neighbors to help the packet's forwarding.

In source routing, a single fixed route of devices is written by the source in the header of the packet. Then, each device in the route forwards the packet to the next specified device until the destination is reached. There are no alternate routes in this mode, so if any device fails on a route, the whole route fails. This is mostly used for network diagnostics.

The authors in [48] present a comparative study between graph routing and source routing. A set of empirical tests on reliability, latency and energy consumption were conducted in a real test bed where WirelessHART was implemented in TinyOS and TelosB motes. The results show that graph routing performed better than source routing in terms of worst-case reliability, although this improvement came at the cost of a higher energy consumption and a longer latency.

The WirelessHART protocol defines three types of graphs: broadcast, uplink and downlink. A broadcast graph links all devices downwards from the gateway to all devices on the network for the transmission of common messages and configuration commands. An uplink graph is a graph that connects all devices upwards to the gateway for transmitting process data. There is a downlink graph for each device in the network in order for the gateway to send unicast messages to each of them [41].

Besides the aforementioned techniques, the WirelessHART specification does not define an algorithm to generate the uplink, downlink and broadcast graphs from the information on the neighbor tables [6]. The implementation of this algorithm is up to the developer. For the sake of comparison with the other technologies, the neighbor table is presented in Table 3.

## 3. State-of-the-Art for WirelessHART Routing Schemes

Since the present paper deals with wireless devices, the network topology is defined by the devices and their neighboring connections within radio range. However, due to fading and interference, many of these connections (links) are unusable. Hence, in the WSN context, routing consists of selecting, given a set of devices, the links to be used.

Routing is very important for network lifetime and performance, since it defines the devices through which the data will pass, something that directly affects the delay, energy consumption and throughput, for example [50].

In this section, we will present the most recent routing algorithms for WirelessHART and highlight their characteristics in order to aid the user's choice for a routing algorithm adequate for his/her needs.

A previous evaluation of WirelessHART routing algorithms was made in [51] in which three algorithms were selected (the Han routing algorithm from [52], the Bellman–Ford [53] and ELHFR [54]) in order to test whether those algorithms were suitable for WirelessHART. From a graph-analysis, the author concluded that the Han algorithm and the Bellman–Ford algorithm were both adequate for WirelessHART networks and had similar performance on the tests (with a slight advantage for the Han algorithm). The ELHFR had worse performance than the others. For this reason, we will only consider the Han algorithm in the present paper.

### 3.1. The Han Routing Algorithm

In [52], a complete solution for wireless mesh networks in terms of routing and scheduling is presented, and in [55], the algorithms were implemented for WirelessHART. In this section, we will only treat the routing aspects. The scheduling aspects will be treated in later sections. The authors in [52] state that most of the algorithms presented in [56–61] focus on identifying multiple node or edge-disjoint paths to improve routing reliability. However, WirelessHART imposes more stringent requirements on routing reliability in order to deal with the harsher and noisier industrial environment. For this reason, the existing literature cannot be directly applied to WirelessHART networks, and new routing algorithms have to be designed.

As a validation method, in [52], a complete WirelessHART communication system was implemented, and the presented solutions were built in the network manager, but the authors abstract the routing requirements presented in the WirelessHART specification. This abstraction is made so that the authors can use their definition of (*k, m*)-reliability, which basically defines the number of entering/leaving edges of the network nodes. Furthermore, the authors assume that the gateway and access point connections are all connected through wires and are reliable, so that the reliability requirements apply only to the wireless devices.

The chosen metric for the greedy algorithms was the average number of hops (*h*), which is calculated over the average of the two best (lower *h*) parents plus one. First, the algorithm builds a broadcast graph by adding one unvisited node per iteration to the “reliable nodes group” (*V_B_*) based on the number of edges from the candidate nodes to *V_B_*. The preference is for nodes with at least two edges to *V_B_*, thus adding the node and the two edges that come from parents with the lowest *h* to the broadcast graph (*G_B_*). Then, those with only one edge to *V_B_* are added.

After building *G_B_*, the algorithm builds the uplink graph (*G_U_*) by inverting the direction of all edges in *G_B_*. Thus, a tree that connects all of the nodes to the gateway is made.

For the downlink case, the paper begins by defining an algorithm that creates a downlink graph for each device in the network, as in the WirelessHART specification. This approach is more complicated (due to the presence of cycles) and is not scalable, since the graph includes multiple intermediate nodes (generating overhead), but cannot reuse the downlink graph information. Then, the paper proposes sequential reliable downlink routing (SRDR), which extends the current downlink route to a sequence of local routes. Then, the local graphs of the intermediate nodes serve as building blocks for longer routes. To support these algorithms, two extensions to the WirelessHART protocol are necessary. First, the algorithm must use the reserved bits (Bits 4–3) of the control byte from the network layer header to indicate the presence of sequence downlink routing fields, storing the ordered graph list in the source routing option field. Second, one must specify how the routing module deals with SRDR, checking, for example, whether the current node is part of one of the intermediate graphs to the packet destination and sending adequate reports to the network manager.

### 3.2. The Joint Routing Algorithm for Maximizing Network Lifetime

**Figure 8:**
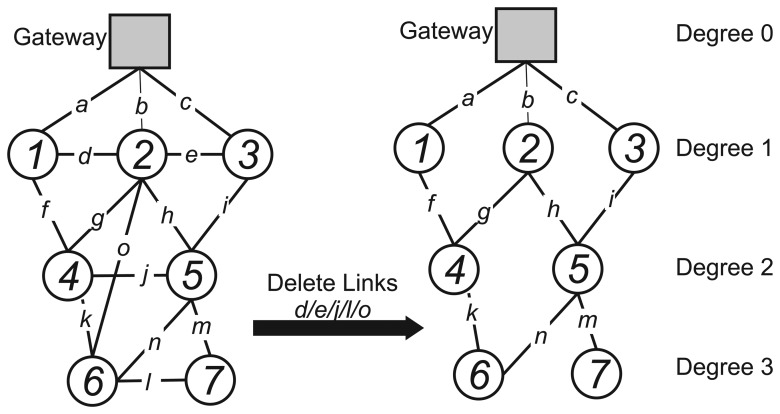
Topology adaptation for the joint routing algorithm for maximizing network lifetime (JRMNL).

A joint routing algorithm for maximizing network lifetime (JRMNL) was proposed in [62]. It is based on the WirelessHART graph routing topology and defines a link cost function for choosing the best next hop node in order to achieve the objective of the maximum network lifetime. Before applying the cost function, the algorithm assigns degrees to the nodes, based on their hop distance to the gateway (the degree assigned to the gateway is zero). Then, the links between nodes of the same degree are deleted, as can be seen in Figure 8.

The cost function for the link between the *i*-th and *j*-th nodes is defined in Equation (2) and is based on the communication load factor of the node (*B_j_*_1_), the energy consumption for that single transmission from node *i* to node *j* (*P_d_*(*i*, *j*)) and the initial and residual energy of the destination (*E_o_* and *E_kj_*, respectively). The ω*_i_* represent the collection of neighbors of the *i*-th node. The *x*_1_, *x*_2_ and *x*_3_ are the positive integers that weight the priority of each specific characteristic.

(2) $$L_{i\to j}=\min_{l\in \omega_{i}}\left({\left(B_{j1}\right)}^{x_{1}}P_{d}{\left(i,j\right)}^{x_{2}}{\left(\frac{E_{0}}{E_{k}j}\right)}^{x_{3}}\right)$$

The communication load factor is defined in [62] and is based on the average transmission interval of each node and its neighbors. The *P_d_*(*i*, *j*) is defined by Equation (3), where *α* is the path loss exponent, *d_ij_* is the linear distance between nodes *i* and *j*, *R*_0_ is the transmission rate and $P_{0}^{s}$ is the probability of successful transmission. The noise terms are set based on complex zero mean Gaussian random variables with variance *N*_0_. This equation was developed by [63].

(3) $$P_{d}\left(i,j\right)=\frac{\left(2^{R_{0}}-1\right)N_{0}d_{ij}^{\alpha }}{-\log\left(P_{0}^{s}\right)}$$

As can be seen, the cost function increases with *B_j_*_1_ and *P_d_*(*i*, *j*), but decreases with *E_kj_*. This means that the algorithm's best case for a node is one for which the communication load and transmission power are minimal and the residual energy is maximal.

The algorithm was validated with a MATLAB® simulation and was compared with the ELHFR (Enhanced Least-hop First Routing) [54] and MPCR (Minimum Power Cooperative Routing) [63] algorithms, obtaining a network lifetime increase of seven- and two-times, respectively. For the average energy consumption, JRMNL was about 4 dBm lower than ELHFR and 2 dBm higher than MPCR.

### 3.3. The Re-Add Routing Algorithm

**Figure 9:**
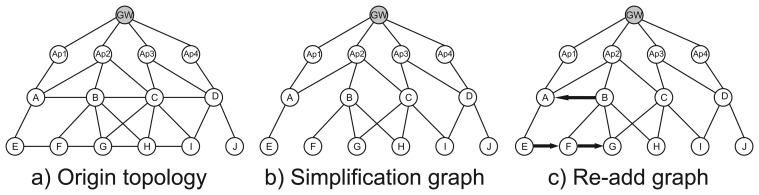
Original topology of the re-added graph.

Re-add is a routing algorithm proposed by [64] that applies a weighted priority function together with an uplink graph construction algorithm to determine the routes for a WirelessHART network. Starting from the original topology, the algorithm assigns degrees (minimum hop count to the gateway) to each node and deletes the links between nodes with the same degree, just as in the JRMNL. However, in order to increase the reliability of the nodes with only one connection to higher degree parents, the algorithm re-adds a link to a node with the same degree. The re-added link must be to the nearest node, in order to ensure the least transmission energy. This process enhances the reliability of the network, because it provides each node with two possible paths (if possible) to forward their packets. The process is illustrated by Figure 9.

Furthermore, the algorithm uses Equation (4) to set the priority of the link between node *i* and node *j* (*P_ij_*). The equation uses the following criteria: link quality (*Q_ij_*), residual energy on the node and the difference between the degrees of node *i* and node *j* (*i.e.*, *D_i_* − *D_j_*).

(4) $$P_{ij}=x_{1}Q_{ij}+x_{2}\frac{E_{i}+E_{j}-P_{d}\left(i,j\right)}{2E_{0}}+x_{3}\frac{D_{i}-D_{j}}{D_{i}}$$

Note that *P_d_*(*i*, *j*) is the same energy consumption for transmission defined by Equation (3) and used by the JRMNL algorithm. Here, *Q* is the ratio of the number of packets received to the number of packets transmitted. However, in order to reduce the cost of the control message for link evaluation, [65] proposed an estimator, a window mean with exponentially-weighted moving average (WMEWMA) and the link quality is calculated by Equation (5).

(5) $${\hat{Q}}_{k+1}=m{\hat{Q}}_{k}+\left(1-m\right)\frac{\text{received}}{\text{received}+\text{failed}}$$

*Q̂_k_*_+1_ and *Q̂_k_* denote the quality of the link at moments *k* and *k* + 1. The values *received* and *failed* represent the number of packets received and lost by a node in a time window *t*. Furthermore, the algorithm uses *m* ∈ [0,1] to control the history of the estimator.

Finally, *x*_1_, *x*_2_ and *x*_3_ are weights that state the relevance of each criteria (quality, energy and degree, respectively). The increase in any of these values represents an increase in the priority of the link. The algorithm uses the analytic hierarchy process as defined in [66] to determine the values of the weights. The process of defining the weights starts from a user-defined matrix that makes a pairwise comparison of the optimization criteria, determining whether each one of them is more, equal or less important than each of the others. Then, a judgment matrix is calculated (see [64] for details), and the weights are calculated by a consistency check.

The author states that simulations were made for validation purposes, comparing it to the Han routing algorithm [52] and the JRMNL [62]. However, the paper only gives the simulation parameters and details for the experiment: no information about the simulator is given. The results suggest that re-add outperforms the other algorithms in terms of reliability and network lifetime. See [64] for further details.

### 3.4. Non-WirelessHARTRouting Algorithms

In order to provide some suggestions and ideas for developing new WirelessHART routing algorithms, this subsection will outline some non-WirelessHART routing protocols. The ideas presented may serve as an inspiration for new WirelessHART algorithms in the future.

For the ISA100.11a technology, [67,68] applied the packet reception rate (PPR) mathematical model in order to define connectivity regions; such regions are classified by the success rate of their routes. Two graphs are built from the topology: the first one is based on the energy consumption of the nodes; the other is latency-based. Then, a scheduling algorithm is applied to the two graphs in order to make a trade-off between energy consumption and latency. The same authors also produced in [69] an industrial cognitive radio sensor network-focused routing algorithm based in ISA 100.11a. The algorithm estimates the maximum throughput for each path and sends the data for most optimal of them.

The algorithm presented in [69] uses a hierarchical cluster-based structure in which stations (called clusters) are responsible for sensing and allocating channels to their neighboring nodes. A cluster head is a special node that uses two separate radio transceivers at the same time, allowing the use of the idle time of the ISM licensed channels. Each cluster can estimate the state of the transmission mean using a method called window mean with exponentially moving average.

The hydro protocol, presented in [70], has the objective of reducing the overhead of the P2P transmissions. Hydro uses the ETXmetric [71] to measure the link quality and adopts three primitives to reach the proposed objective. The first primitive determines the formation of a distributed DAG (directed acyclic graph); thus, each node must have a list of default route devices that will lead to the border routers. The second primitive is the global topology construction that defines a topology report, which is piggybacked onto the data traffic. This report contains information about the best neighbors in the list of default routes, so that a graph can be built. Finally, there is a centralized route installation, which determines whether routes should be installed/updated in the devices by the border routers by a route install message.

**Figure 10:**
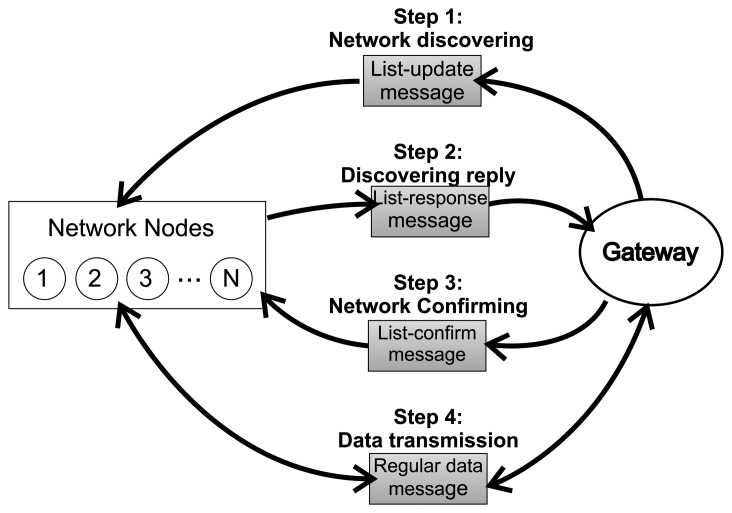
REALFLOW proceeding summary (adapted from [72]).

Although the work presented in [72] is very interesting due to the focus on the challenges of the IWSN, the present work does not conform to the WirelessHART current specification, becoming more of an option for future updates for the technology. Thus, we will emphasize it in this subsection due to the innovative approach presented. The work in [72] proposes the REALFLOWprotocol, which focuses on industrial wireless sensor networks. The protocol includes a routing algorithm, which provides multipath diversity (in order to increase reliability), real-time performance, tolerance to sudden changes in the topology and a workload division among the devices in order to allow the network manager to save processing time in routing information calculation. In order to achieve the aforementioned objectives, the authors divide the solution into two parts: a routing establishing and management part and a packet forwarding part. The relationship among those parts and the network structure are illustrated in Figure 10.

Steps 1–3 in Figure 10 represent the three control messages used for the routing establishment and management. The networking discovery is performed by the list-update message, which is broadcast from the gateway to the devices carrying information about signal levels, hop count and the previous node address. When a device receives a list-update message, it updates the parameters contained in the message and rebroadcasts the updated message as needed in order to reach the whole network. The accumulation of signal levels determines the most reliable routes; the hop count avoids endless rebroadcast (the packet is discarded if the hop count is greater than the node depth); and the previous node address keeps track of the path covered by each packet. After receiving the list-update messages, the nodes of the networks report to the gateway with a list-response message. This message contains information about the neighboring nodes (siblings, parents and children) and the next hop from that device. The gateway uses this information to build the network topology. Each list-update message has a sequence number attached to it in order to avoid duplicate packets. Finally, the gateway sends list-confirm messages to all nodes with the confirmation of related node lists and scheduling decisions. The aforementioned packet forwarding part consists of a set of criteria that restricts the flooding of messages, in order to save the network resources. The first one is the definition for each node *n* of a local related node list *L_n_*, which contains a list of addresses from the nodes considered related to *n*. A node *x* is considered to be related to node *n* if *x* is involved in forwarding packets between *n* and the gateway. Thus, the criteria define that once a node *n* receives a packet, the node will only consider forwarding this packet if the source or destination address from the received packet belongs in *L_n_*.

The second one consists of marking the packets with a unique identifier. This identifier comprises the sequence number of the packet and the source address of the current node. The identifiers of the packets are stored in each node using a history table H, and once a new data packet is received, the incoming identifier is compared to those present in H. If the identifier is already present in H, the packet is a duplicate and must be dealt with accordingly. Finally, the last criterion is the packet age. Outdated packets can be of limited use in industrial networks; thus, each packet carries an age value; this age value is compared to the deadline value for that packet before being sent out. If the age value is greater than the deadline value, the packet is discarded in order to avoid buffering in the intermediate nodes.

The drawback of the routing algorithm presented by the authors in [72] is the increase in the energy consumption due to the use of the flooding algorithm and the focus on reliability and real-time performance.

#### 3.4.1. Comparative Analysis

In order to obtain relevant information about the described routing algorithm proposals, we will discuss the items described in the list below. Furthermore, Table 4 presents an overview of the analyzed proposals.

- Objective: Different routing algorithms are developed to solve different kinds problems or to improve specific characteristics in a network. This item shows the goal that the authors intend to achieve with the developed algorithm proposal.
- Metric: The metric consists of which characteristics were chosen to guide the routing decisions of the algorithm. This is an important matter, since the metrics chosen will have a direct impact on the performance of the network and on whether the objective of that algorithm will be achieved.
- Implement uplink, downlink and broadcast graphs: The three graphs presented in the WirelessHART specification have distinct features; thus, it is to be expected that some algorithms deal differently with the construction of each graph. This item identifies with which types of graph the proposal deals.
- Uses node history: This item identifies whether the algorithm uses the past states (e.g., the energy levels or the success rate) of the node in order to make the routing decisions.
- Implementation and validation: Different proposals vary in how they are conveyed to the reader, ranging from a description of the policies to be used, up to describing the steps in an actual algorithm. This item describes how the authors present their proposal and also describes the means of validation (e.g., simulation, testbeds).

Objective: In terms of the objective, all of the proposals have the same concern for maximizing the network lifetime, which is relevant in a battery-powered IWSN environment. However, the most recent re-add [64] proposal includes in its objectives the robustness of the network, reaffirming the WirelessHART robustness feature.

Metric: The comparison presented in Table 4 shows the concern for mixing different metrics in order to balance obtained results. Furthermore, the JRMLR and re-add proposals present the uses of weights to tune the function and prioritize a specific characteristic.

Implement uplink, downlink and broadcast graphs: Of the proposals, only the Han routing algorithm presents a specific treatment for each one of them, meaning that it is nearer to an actual network manager implementation that can fulfill the WirelessHART specification.

Uses node history: As a dynamic environment, a WSN is subject to variations in its communications, due to issues like interference, for example. These variations could imply anything from a small bit loss up to the incapacitation of whole channels, lasting from milliseconds to days of interference. Such a situation should be relevant to the routing algorithms in order to provide an adequate response to an interference situation. The use of the node history by the re-add proposal can be seen as a way to avoid having small interferences cause changes in the routing and overhead that is propagated with the new configuration, consuming network resources.

Implementation and validation: Of the analyzed proposals, all use simulations for their validation. However, the Han routing algorithm has a more consistent one, due to its implementation by a different group in [55] and also by the presentation to the community of an actual algorithm.

## 4. WirelessHART Scheduling Schemes

The literature contains the results of research into scheduling for wireless sensor networks. For example, we can cite algorithms, like [73] (which is based on the distributed coloring of the nodes), the QoS-supportive distributed scheduling algorithm for real-time applications (D-SAR) in [47] and the rapid response oriented source-aware SAS-TDMA in [74]. Furthermore, delay and jitter minimization solutions are presented in [75], respectively, [76]. [77] presents a survey of TDMA-based MAC protocols for WSN communications, which includes orientations for scheduling proposals. Although this literature is relevant, it was not specifically developed for the WirelessHART technology and its requirements.

In order to attend to the demand for a WirelessHART-focused survey, in this section, we will focus on the main scheduling proposals for WirelessHART.

### 4.1. The Dang Algorithm

The work in [78] presents a scheduling algorithm for the graph route technique, scheduling end to end communication resources, with the intention of meeting the WirelessHART specification. The algorithm deals with multiple publish rates, thus dealing with multiple superframes, and seeks to order the scheduling of the links according to the connection sequence indicated in the graph tables.

The algorithm schedules each superframe, from the superframe with the shortest length to the superframe with the largest length. It is worth noting that the devices that are attributed to a given superframe have the same publish rate, and this common publish rate defines the length of that specific superframe. Thus, the fastest publish rate devices have priority over the slower ones. A table is used to store the schedule, which is called SlotNumber, and relates the slot time with the channel used, avoiding time slot and channel conflicts among the transmissions.

In order to schedule the communication over the actual sequence of devices along the path to its destination, a variable named LastSlot is used. There is a LastSlot variable for each device, and it stores the last slot time in which the device was scheduled. When the next transmission is to be scheduled, it will be scheduled on the next available slot after the LastSlot (first fit technique).

The algorithm uses the retransmission scheme based on the scheme proposed by the WirelessHART specification as described in Section 2. The algorithm uses two Booleans to control whether the link being scheduled is a reservation for a retransmission: *First*, which indicates whether the transmission is an initial one or a retransmission, and *FirstChannel*, which stores which channel was used in order to schedule the retransmission in a different channel. Transmission trough different devices from the main route should be directly in the superframe.

In order to validate the proposed scheduling, the paper did an analysis of the proposed scheme and compared it with single, double and triple redundant routes schemes. Unfortunately, no testbed or simulation results were presented.

### 4.2. The Zhang Algorithm

The work in [79] proposes a scheduling algorithm that uses graph coloring for the graph routing only and aims to optimize latency and provide high reliability. The edges represent the links from the network, and their color (an integer number) represents the slot time in which that link should be scheduled. Two adjacent edges must not have the same color. Furthermore, a channel number is used to differentiate links with the same color (thus being in the same slot time). All edges in the network must be dyed in at least one color.

In order to provide reliability, the algorithm uses ORMGR [80] to determine an optimal transmission path and uses a retransmission policy. This policy considers two data retransmission opportunities on the optimal route. Edges on the optimal route have priority for being dyed and have two colors along with different channels. Thus, if the data transmission fails through a link on the best route for the first time, then the device sends the data through another channel to the same neighbor. The second retransmission occurs on another link. However, these alternate routes do not have an additional slot arranged in order to improve the network throughput.

In the algorithm, the graph is traversed from source to destination coloring (staining) the edges based on the source's degree *d*. When the links related to the nodes of degree *d* are scheduled, *d* is decreased and the algorithms proceed until the last degree (zero, from the gateway) is reached. The algorithm builds two groups of devices, E and E'. E is the group of all links in the network, and E' is the group of stainable devices in an iteration. Group E' is formed by the links that connect nodes from degree d and their upper or lower-layer nodes. A node can appear at most once in E', and dyed links of the optimal route should be included. In each iteration, if there is no non-dyed edge *e* from the optimal path, the variable *d* is incremented, and the algorithm moves to the next degree. The edges are dyed at the end of each iteration, and the available channels are distributed to the allocated links. The colors are determined by a variable *s*, which is incremented at the end of each iteration.

In that paper, there are tests for validation purposes and a comparison with the algorithm presented in [78], but no further details about those tests are revealed. The paper claims that its algorithm has a better success rate than both the algorithm proposed in [78] and a non-retransmission alternative. This advantage grows with increasing network depth.

### 4.3. Conflict-Aware Least Laxity First Algorithm

The work in [81] presents a proof for the NP-hardness of the scheduling problem in WirelessHART technology, and two scheduling algorithms to solve this problem. The first is an optimal branch and bound-based scheduling algorithm, that uses a search tree, so as to guarantee that a schedule will be found whenever a feasible one exists. The algorithm metric is the laxity of a packet, which consists of the remaining time slots minus its remaining number of transmissions in the transmission route.

Although the algorithm is optimal, the execution time needed limits the application of the algorithm in a WirelessHART network due to the dynamic nature of such an environment. In order to attend to this requirement, the authors proposed a second algorithm based on the idea that the algorithm must be cognizant of conflicts between transmissions, the conflict-aware least laxity first (C-LLF) heuristic algorithm based on the least laxity first, which has been used successfully on wired networks. The conflict-aware laxity metric consist of considering the length of the time windows in which the transmissions can be scheduled and the potential concurrent transmission windows that would cause conflicts.

The windows are determined by the values of the release time (*r*) of a transmission τ*_k_*, which is the first slot in the superframe in which the transmission can be scheduled, and the deadline (*d*) of that packet to reach its destination. The release time and the deadline are respectively affected by the number of hops to the transmission link (*pre_k_*) and the number of hops left from the link to the destination (*post_k_*). For example: if a transmission τ*_k_* is three hops away from the destination, the deadline for this transmission to happen is the deadline of the packet to be delivered minus three slots.

The scheduling algorithm starts from a pool of all transmissions Γ to be scheduled and begins allocating from the first slot time to the last (the variable *s* goes from zero to the size of the superframe). The algorithm runs until it schedules all transmissions (*τ_k_*) by selecting a group (Released(s)) of transmissions that have a release time equal to *s* (the current slot time being allocated). From this group Released(s), the algorithm gives priority to those transmissions with the closest deadline and the bigger number of conflicts in their lifetimes. When a transmission is scheduled, all conflicting transmissions are removed from the Released(s) group to be scheduled in another slot. The algorithm tries to allocate all transmissions from Released(s) in the available channels of the slot time before proceeding to the next slot. The algorithm ends in success if it manages to schedule all transmissions from Γ, but ends in failure if a transmission misses a deadline and is unschedulable.

To validate the algorithm, the paper describes both simulation and testbeds comparing the proposal to a set of baseline real-time scheduling policies. The three metrics used were:

- Schedulable ratio: the percentage of test cases for which an algorithm is able to find a feasible schedule.
- Buffer size maximum of packets buffered at a node when transmissions are scheduled.
- Execution time: average execution time to generate the schedule for the packets generated in a superframe.

The proposal outperforms the other policies in most parts of the tests, except for the execution time, which grew more than the others with an increasing number of nodes in the network. Nevertheless, the calculation time obtained was around 2.5 s, which is a reasonable computation time for a WirelessHART network. For more detailed results, see [81].

### 4.4. The Han Algorithm

The algorithm presented in [52] uses a fastest sample rate first (FSRF) policy to schedule the network transmissions. The scheduling is based on the reliable graphs presented in Section 3.1 and uses a recursive function to allocate the links in the form of a global matrix 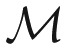. This recursive function allocates the nodes from source to destination using a depth first search algorithm (DFS). The matrix **
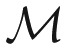
** represents all of the allocations for channel/slot time for each link; its dimensions are given by the number of channel (16) *versus* the size of the lengthiest superframe. The superframe 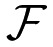
*_i_* represents a group of devices with sample rate *r_i_*. The variable *l_i_* represents the length of the superframe 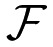
*_i_*.

**Figure 11:**
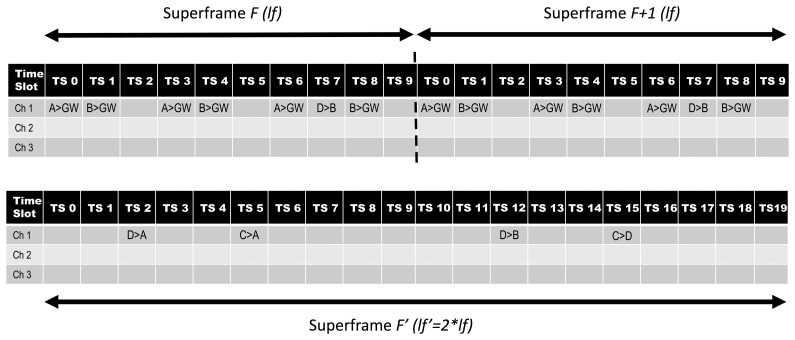
Superframes 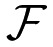
′ and 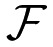 (Figure from [51]). Reproduced with permission from Victor Dickow, “Avaliacao de Algoritmos de Roteamento e Escalonamento de Mensagens para Redes WirelessHART”; published by UFRGS, 2014.

Generally, each device is several hops away from its destination. If the network manager allocates all of the resources from the multiple paths into the scheduling, the schedulability of the network schedule would degrade rapidly In order to avoid this, the algorithm uses a parallel $F_{i}^{\prime }$ superframe, with length *l_
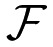
′_* = 2 * *l_
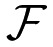
_*, to accommodate those transmissions from nodes with two possible routes (see Figure 11). This occurs by reducing the transmission rate by one-half and determining an offset that positions the links in a communication pattern identical to the original, but by different routes. It is worth noting that reducing the publish rate means, for example, that a device that publishes once per second will be equivalent to a device that publishes once every two seconds.

The allocation is divided by the type of link to be scheduled. The links can be an uplink or downlink and exclusive or shared. An exclusive link is one occupied by two devices for dedicated communication. Shared links allows multiple devices to compete for transmitting to the same device simultaneously. The division can be seen in Table 5.

Shared links may be used by stations for retries, thus enhancing the reliability of the network. If an attempt to schedule a link fails, the network manager sends a message informing the requiring node of the lack of bandwidth.

The scheduling implementation was validated with a simulation and presented an improvement, mainly in the network utilization metric, while maintaining a scheduling success rate near 100% for lower sample rates.

### 4.5. The Zhang Policy

**Figure 12:**
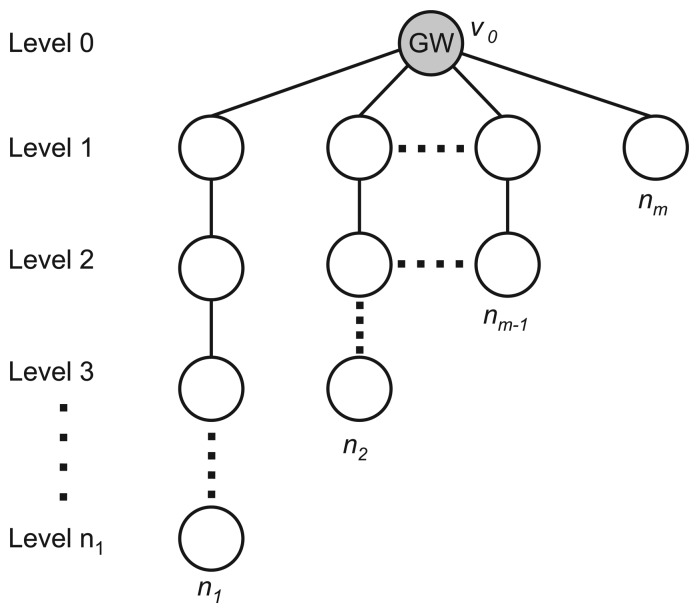
Multi-line topology example.

The work in [82] establishes the lower bound for the convergecast time, establishes a lower bound for the number of channels for time-optimal convergecast and proposes two scheduling policies based on the optimal scheduling for line topologies proposed in [83]. It is important to note that no actual algorithm was proposed and that no redundancy technique was adopted. The proposed policies apply to multi-line topologies like the one presented in Figure 12. It is worth noting that the main issue addressed by this proposal is the fact that the last hop to the gateway is a limitation on the system, since all of the packets must pass through this hop, and thus, the half duplex radio of the gateway limits the flow to one packet per slot time.

The first policy guarantees a time optimal convergecast, sustaining the established lower bound for the size of superframe needed to allocate all transmissions. However, this policy is not efficient in terms of channel usage. In order to achieve something near-optimal in that way, the second proposed policy uses the established lower bound to the number of channels, although this bound is not always achievable. The second policy (time- and channel-optimal convergecast policy) is a heuristic approach that is based on two steps that should be applied to each time slot.

The first step is called connectivity keeping and traverses the levels of the topology from the gateway to the leaf nodes and prioritizes the scheduling of links that avoid the so-called schedule holes. The schedule holes are those that have a slot time where the gateway does not receive a packet, while the network still has packets; in other words, the transmission opportunities to the gateway are underused. This situation causes delays for all of the network schedule.

If the number of devices scheduled in connectivity keeping is not the maximum that this slot supports, the policy called deadline-based scheduling should be applied. This policy organizes the devices that can be scheduled for transmission in that slot time and sorts them in non-decreasing order, giving scheduling priority to those with tighter deadlines. The hop count is used as a secondary sorting parameter.

A heuristic algorithm based on the presented time- and channel-optimal convergecast policy was implemented in a MATLAB® simulation and in a Contiki OS Java (COOJA) simulator [84]. For the MATLAB® simulation, the proposal obtained a schedule that used at most one slot time more than the established lower bound schedule length. Of the 10,000 runs, only two did not reach the lower bound for the convergecast time. The test on the COOJA simulator showed that the scheme can collect data from 34 devices divided into six lines (the lines have, respectively, 10, 8, 6, 4, 4 and 2 devices) in 0.4 s for a 125-byte payload.

### 4.6. Non-WirelessHART Scheduling Algorithms

As we did in Section 3.4 for the routing algorithms, in this subsection, we will present some of the newest scheduling algorithms for WSN in order to provide some ideas for developers that intend to build new scheduling algorithms for the WirelessHART technology.

The work in [85] presents the D-MSR (distributed network management scheme for real-time applications), a network management system that provides real-time, reliable communications and fulfills the throughput requirements of industrial control applications. For scheduling purposes, the system adopts the distributed scheduling algorithms for real-time applications (D-SAR; see [47]). It is worth noting that the D-SAR use concepts based on the ATMnetworks [86]. D-SAR uses a set of messages that traverses from the source to the destination based on the routes from the routing setup. Each device includes parameters, such as a list of suggested common unused timeslot-channel cells for further communication with the next hop, a destination address, traffic ID and the timeslot-channel cell selected on the previous hop. With this information, the devices organize their own schedule matrix and pass the message forward to the destination. It is also important to point out that D-MSR uses the virtual circuit technique in order to provide real-time communication.

The work in [87] proposes a service-oriented and Markov random field (MRF)-based scheduling algorithm for WSN, called MRF-based multi-service node scheduling (MMNS). It is worth noting that the proposed algorithm is a heuristic solution. Its objective is to manage devices that are equipped with multiple sensors (for example, sound, motion and temperature), thus providing various sensing services from the same node. The proposal intends to arrange resource sharing among multiple services in an energy-efficient way. The MMNS algorithm is based on two other algorithms developed in [87]: the multi-service data denoising (MDD) algorithm and the representative node selection and service determination (RSD). The first one has the objective of minimizing the noise level of the sensed data. The later intends to detect representative nodes of the network and the services that they provide.

The work in [88] differs from the other papers in this area since it presents two scheduling methods for shared timeslots in ISA100.11a networks. The first one, traffic-aware message scheduling (TAMS), uses traffic information to define groups of devices and decides in which cycles the groups will attempt to transmit in order to use the bandwidth wisely by avoiding collisions in every cycle. The second algorithm is the contention window size adjustment (CWSA) algorithm, which also uses traffic information to adjust the size of the contention window (fixed at 192 microseconds by the ISA100.11a specification, regardless of the traffic) when the probability of a collision in a timeslot exceeds a threshold.

The algorithm developed by [76] has the objective of reducing the jitter provoked mainly by multiple arrivals of each packet through different paths and by the drift of the starting/ending slot position in a superframe. In order to do this, the algorithm classifies the transmissions into tasks that group the transmissions so as to take the data from the source to the destination. After the tasks are defined so as to avoid conflicts, the schedulability is guaranteed by giving priority to tasks based on the information about their deadline and the number of hops.

The work in [75] describes a method that finds conflict-free TDMA schedules and minimizes scheduling delay in multi-hop TDMA wireless networks. The solution is divided into two parts. The first one consists of finding a transmission order that satisfies the precedence requirements for the communication. This solution uses integer programing in order to find the transmission order that minimizes the maximum scheduling delay. The second part is to provide the transmission order together with a conflict graph (see [89,90]) to the scheduling algorithm (the one used was a Bellman–Ford algorithm), so that the transmissions can be accommodated in the schedule without conflicts.

The work in [91] presents a new medium access protocol-based on the WirelessHART and ISA100.11a that focuses on addressing IWSN critical traffic and latency issues. As a main feature, it uses the initial and final parts of the traditional time slots, so that higher-priority transmission may signal to devices that a higher-priority transmission will occur; thus, devices should hold low-priority transmission scheduled for that time slot. This scheme allows higher-priority packets to hijack the bandwidth from lower-priority packets during the network operation. Although this technique is not applicable to the current WirelessHART specification, the signaling scheme may be used in future installments of the IWSN standard specifications.

The work in [92] combines traditional TDMA with slotted aloha in order to produce an optimal schedule for a single hop delay-constrained sensor network. This algorithm aims to improve reliability, while avoiding the drawback of not providing efficient usage of resources, a common situation in traditional TDMA schemes. It improves the works presented by [93,94] by using slotted aloha instead of CSMA, thus avoiding the variable delay drawback introduced by exponential CSMA backoff. Thus, two algorithms are presented: one that computes an optimal schedule and a heuristic algorithm that manages to compute solutions with a success probability close to the optimal solution, but with much more modest computational complexity. In summary, the strategy consists of the following: based on a Bernoulli loss model, each transmission has a success probability *p_i_*, and from that, the algorithms distribute the transmission in the schedule in order to maximize the success probability of the schedule. Each time slot can be a dedicated slot, where only one transmission is scheduled to occur, or a shared slot. In a shared slot, the devices scheduled to occur may attempt a transmission using the slotted aloha scheme if they have failed their respective dedicated slot attempts to transmit. The optimal solutions consist of trying all of the possible combinations in order to maximize the success probability of the schedule. The heuristic solution consists of a greedy algorithm that chooses the next device(s) to be scheduled in order to maximize the probability of the controller receiving one more packet in the *k* + *1* step based on the already defined previous *k*-th step. The numerical simulation results of [92] show that their scheme outperforms the traditional exclusive slot TDMA approaches, thus making the use of shared slots a relevant topic for future works.

The paper [95] focuses on integration of security and safety in IWSN and proposes a full framework for providing secure and safe communications for the network. As the focus of our present work is routing and scheduling, it is worth noting that the paper [95] expresses concern about the focus of the current WirelessHART implementations on the uplink transmissions over the downlink transmissions and how this would affect the safety of WirelessHART-based distributed control systems (DCS). For example, a delay in delivering a turnoff signal sent from the manager to an actuator in a critical state may provoke safety issues. The authors state that in order to achieve good results, all of the set points for the actuators must be distributed for the devices in the same cycle, and jitter and delays should be reduced to a minimum. Thus, they propose the implementation of a new WirelessHART command that the control application can use to set periodic transmissions up to the actuators (downlink transmissions).

The work in [96] has to be objective to maximize reliability for end-to-end packet delivery in IWSN. The paper organizes the network topology into disjointed sets of hypernodes. The hypernodes consist of a set of devices that connect directly among each other, thus forming a complete graph. The logical organization of a set of hypernodes forms a hypergraph. Finally, in terms of scheduling, the paper proposes the division of the scheduling into two parts. The first one is the dedicated scheduling, which decides the quantity of slots that should be allocated to each hypernode in order to fit the periodic packet transmissions between the devices and the respective destination. The second scheduling scheme, called the “shared slot scheme”, allows the hypernodes to use remaining time slots from successfully delivered transmissions in order to enhance the flexibility and reliability of the communications. It is worth noting that the proposed scheme supports both single-path and the any path routing introduced in [97].

Although the work presented in [98] is not a scheduling algorithm, it deals with the flow priority assignment issue in WirelessHART networks. This issue consists of assigning priorities for real-time flows, so that those flows can meet their deadlines, and also works in alignment with schedulability tests, so that the networks can support effective network capacity planning and efficient admission control of devices and topology adaptations. As the optimal priority assignment is the NP-hard problem, the paper first proposes an optimal priority-assigning algorithm based on local search for any given worst-case delay analysis. However, for the sake of efficiency, the paper proposes a heuristic search algorithm for priority assignment.

The paper in [99] addresses the problem of single-hop networks with heterogeneous traffic with packet delay constraints. The objective of the paper is to provide optimal time slot allocation and to schedule the packet transmission. The author divides the solution for this problem into two parts. The first part is called the “subperiod slot allocation problem”, which consists of defining how many slots each device will have available for transmission/retransmission based on the success probability of each link. This part of the problem is NP-hard and was modeled as a nonlinear integer programming problem. Thus, the authors propose converting the problem into a linear integer programming problem and then present a polynomial time algorithm to compute the optimal solution for it.

The second part of the problem is a matter of allocating which slots are allocated to each device in order to deal with the delay restriction defined by the publish rate of each device and how those transmissions relate to other transmissions from different devices. This is accomplished by allocating the number of slots defined for each device in a time subperiod defined by the report period for that device and then combining those subperiods into a hyperperiod. This hyperperiod has the duration of the least common multiple of the report rates present in the network in order to accommodate multiple subperiods without fractionating. However, the technique is not suitable for WirelessHART, because it focuses on single-hop wireless networks and, thus, differs from the multi-hop nature of the WirelessHART network. Although the system model proposed is compliant with the WirelessHART specification, it could also be applied in the WIA-PA and ISA100.11a single-hop cluster structures.

### 4.7. Comparison of the Scheduling Protocols

In order to compare the previously presented algorithm proposals for the WirelessHART technology, we present in this section a set of items to be compared between the algorithms. Those items are:

- Objective: Developers of an algorithm may have different applications, situations and needs, which require different solutions. This leads to the development of a variety of different algorithms. Thus, the objective states what is the main thing that the proposal tries to improve when used in a network.
- Metric: The algorithm needs to use parameters in order to make choices that will build the schedule and prioritize the developer's objectives for the algorithms. The metrics are those parameters (e.g., degree, deadlines) that are used to determine the links and channels that will be scheduled.
- Multiple superframe: The WirelessHART specification defines that multiple publish rates must be supported in order to deal with different types of devices and processes. Devices with the same publish rate must be grouped in the same superframe. The multiple superframe item identifies whether the proposal explicitly deals with multiple superframes.
- Redundancy: The proposals may view the scheduling problem in two different ways: one is to view the problem as a pool of transmissions that must be allocated in a schedule; the other is to see a group of end-to-end transmissions that should have each of their forming hops scheduled and provided with redundancy. The redundancy item describes whether the proposal uses or suggests a redundancy method.
- Implementation: Different methods can be used in order to validate a proposal. This can range from simpler approaches, like an analysis of the solution to a full testbed implementation in real devices. Furthermore, there are cases where the proposal presents an actual code for the solution, but there are cases when just the policies that should be used are presented. In order to clarify those questions, the item implementation shows whether the paper presents actual pseudocode for the algorithm and which validation methods were used (e.g., testbed or simulation)
- Flow differentiation: The WirelessHART specification classifies the information flow to and from devices to the gateway (sink) as being an uplink and downlink, respectively. As these flows have different characteristics, the flow differentiation item identifies whether the paper specifically treats the uplink and downlink flows of the WirelessHART network.

Table 6 presents the characteristics of each proposal for each item. The intention is to identify which trends prevail for the scheduling in WirelessHART, thus giving direction for further improvements in the field.

Objective: We can see that there are two main trends for the objective. The first includes those which are mainly concerned with filling up the scheduling, like the Dang algorithm [78] and C-LLF, as this is a complex problem to be solved by itself. The second trend is formed by those algorithms that are proposed in order to specifically improve specific characteristics, like latency, reliability, time and channel usage.

Metric: A balance between the three metrics, degree (this is the same as a hop count), deadline (laxity involves deadlines upon calculation) and publish rate, since it can be seen that all proposals use one or more of these. This reveals a clear trend for those metrics, but leaves space for new ones, like robustness.

Multiple superframe: A concern for multiple superframes is only explicit in the proposals of the Dang algorithm and Han algorithm, which use publish rate as a metric for decisions. The others proposals did not specify how to deal with the issue and leave that for the implementation of the manager to solve.

Redundancy: Three proposals presented a solution with a concern for redundancy, revealing themselves as more complete solutions. The solution presented in the Han algorithm [52] has a particular feature: this proposal uses shared slots for possible retries; thus, the devices that need a retry compete for the possibility of using the transmission medium.

Implementation: Most of the proposals implement their ideas using simulation, which is reasonable for reasons of cost and reliability issues, as can be seen in [100]. The C-LLF and Zhang algorithms use testbeds, but the latter does not provide more details on it. The most deviant form of validation is the analytical comparison presented for the Dang algorithm, which uses calculations based on the number of hops and the reliability of the links.

Flow differentiation: Only the Han algorithm [52] implements the flow differentiation feature in order to define each region of the superframe that would be occupied by each flow, as presented in Table 5. All other proposals deal with the flows indiscriminately.

## 5. WirelessHART Simulators

When developing a new protocol or algorithm for WSN, it is very important to measure the performance of the proposal in order to determine the most adequate scenarios for it, its strong points and downsides. It is also important to compare the proposal's performance to that of other already implemented solutions. To do this, there are two possibilities: the implementation of a testbed with real devices and computer simulation. Although testbeds provide realistic results, the costs and the complexity of the test environment, mainly in its early development stages, may make that option not viable. Thus, computer simulations can be used as a low-cost and scalable solution for testing new proposals before investing resources into real networks. Furthermore, simulations may also be used to guide the choice of users that are trying to decide on a WSN technology. In face of this situation, we will present in this section a description of the main available WirelessHART simulators, their features and where to find them.

The COOJA (Contiki OS Java) simulator is a Contiki OS simulator. The Contiki OS is an operating system (OS) developed to run on devices with limited resources (for example, battery, memory, processing), like sensors [101]. As the COOJA simulates each node, like devices that run an actual OS, the code developed for the real OS can be used in the simulator. The main goal of the COOJA is to provide extensibility to the simulation. To achieve this, the simulator implements two different structures: plugins and interfaces. Plugins are registered often at start up before being used and allow interaction with the simulator. Interfaces interact with the sensor nodes, simulating hardware peripherals, or monitor interest values. In order to simulate WirelessHART, [102] developed a solution, which is to add a WirelessHART-enabled intermediary device operating at the physical and data link layers, which can handle the time-critical tasks of the protocol. Furthermore, with regard to the Zhang policy, [82] claim to have used COOJA, but few details about the simulator were provided. Finally, it is worth noting that COOJA is open source software.

Network Simulator 2 (NS-2) is an open source discrete event simulator for network research. The first NS began as a modification of the REALNetwork Simulator in 1989. Nowadays, the NS development is maintained by a collaborative effort of diverse institutions and researchers. The simulator provides support to TCP simulation, routing and multicast protocols in wireless or wired networks, local or satellite communications [103]. The NS-2 uses two programing languages: C++ is used to code the protocols to be used and the interpreted programming language OTCL (Object-oriented Tool Command Language) to configure the simulation parameters. In order to provide a better understanding of the simulations, NS-2 provides Network Animator (NAM), a tool that animates the traces of a completed simulation. A WirelessHART implementation for the NS-2 can be found in [55].

Although NS-2 is a very popular simulator, it has issues with regard to scalability, memory use and simulation time [104]. Furthermore, it has an outdated documentation and and great abstractions in the lower layers [103]. In order to address these issues, its developers have started the open source Network Simulator 3 (NS-3) project, a natural successor to NS-2. The new simulator uses an updated, modular and object-oriented structure with a vast available documentation. The modular structure is such that modules can be implemented in way that they can be reused in different technologies modules. The NS-3 will work with the C++ language and, optionally, Python scripts. A WirelessHART module for NS-3 with a focus on modeling the lower layers, providing energy consumption and independent error rates for each link, can be found in [100,105].

MATLAB® is a well-known (proprietary) tool for the scientific community, providing tools for a wide range of fields, from neural networks to aeronautics. Therefore, it is a natural platform for the development of network simulation modules. The work in [106] presents a modification in the wireless network block of the TrueTime simulator in order to simulate WirelessHART networks. The modifications were made using some C++ functions and MATLAB MEXinterfaces, and the simulations are used using Simulink's block language. It is worth noting that the JRMNL algorithm presented in Section 3.2 was validated using MATLAB® [62], but a specific simulation module for the platform was not developed.

Another approach for the TrueTime platform was implemented by [107] aiming at the application levels, mainly control loops. The module provides information about possible errors in data flow dependencies and network access conflicts. However, the implementation does not support multiple hop communication and utilization of the 15 channels defined by the WirelessHART specification [6].

OPNETis a proprietary discrete event network simulator that adopts a hierarchical structure divided into three layers (network, node and process) that is intended to be parallel with the structure of actual communication networks [108]. One WirelessHART implementation for the OPNET platform is presented in [109]; it tries to build an entire WirelessHART network stack. This network model can be used as a simulation platform to test resource scheduling algorithms' performance and internetworking protocols. Another implementation can be seen in [110], where the author claims it to be a “primary implementation of the medium access control layer of WirelessHART” and proposes the improvement of the shared slot access method by using slotted CSMA-CA instead of the existing aloha. The language used in OPNET is a proprietary block diagram language with the possibility of event scheduling. It is worth noting that nowadays, OPNET is available as a part of the Riverbed Network Simulation Software [111].

For the sake of a better understanding, Table 7 will outline some key points of the aforementioned WirelessHART simulation modules.

## 6. Conclusions and Future Research

This paper presented a set of the most recent routing and scheduling algorithms for the WirelessHART technology. Through the discussions of routing topics, we can verify that this is a rich field, in which the variety of the algorithms and solutions is growing along with the number of applications where WirelessHART is applied. For example: monitoring applications can use mesh networks in order to improve their reliability through the use of spatial redundancy (multiple paths), and control applications can use temporal redundancy (multiple copies of the transmitted data). For other applications, there is also the possibility of using a hybrid solution.

As indications for future research in this area, we can suggest studies of the metrics used so as to include new characteristics (e.g., robustness), using the ideas from the weighted functions of the routing algorithms in the decision making for the scheduling algorithms and developing an automatic adaptation of the function's weights based on the network life cycle. Based on the fact that the majority of the scheduling algorithms presented use dedicated slots for scheduling, we can also propose for future study the usage of shared slots in scheduling (as in [92]) in order to improve reliability. Furthermore, it is worth noting that the heterogeneous traffic issue presented in [99] has still not been fully explored in the literature, and thus, is an interesting topic for future works. Another possible future study that we can point to would be about the influence on the network's behavior of the concentration of the transmission in specific regions of the superframe.

A point that could be more explored in the new technologies presented is the matter of their scalability. The performance of bigger networks is more affected by the overhead of the routing and scheduling algorithms; thus, scalability is a key issue when choosing algorithms for a WirelessHART network.

One difficulty encountered was how to compare the different technology implementation methods, even though all of them used simulations as a validation method. One possible question is whether it is possible to compare those simulations fairly and how to do so. One possible solution may be a simulation tool that would submit all of the protocols to a standard test that would present the same conditions for all of the tested protocols.

Finally, as the standard does not define routing and scheduling algorithms and noting the plurality of solutions presented, we can conclude that the development of optimal solutions for the diverse scenarios of the IWSN will demand more time despite the efforts made so far.

## Figures and Tables

**Table 1:** Main features present in the IEEE 802.15.4 physical layer. O-QPSK, quadrature phase shift keying; BPSK, binary phase shift keying; ASK, amplitude shift keying.

**Features**	**IEEE 802.15.4 Physical Layer**
Frequency bands	868 MHz (1 channel)
	915 MHz (10 channels)
	2450 MHz (16 channels)
Channel width	2 MHz
Throughput	250 kbps, 40 kbps, and 20 kbps
Modulation	O-QPSK, BPSK and ASK
Communication range	100 m to 200 m
Transmission power	0 dBm

**Table 2:** WirelessHART scheduling constraints (table adapted from WirelessHART specification [6]).

**Features**	– The maximum number of concurrent active channels is determined by the number of enabled channels and limited by black-listing. – No device can be scheduled to listen twice in a slot. – More than one device can transmit to the same device. A broadcast link and dedicated links to each of the listening devices can coexist. – On a multi-hop path, early hops are scheduled first. – The supported update rates are defined as 2*^n^*, where *n* is a positive or a negative integer. For example, update rate selection can be 250 ms, 500 ms, 1 s, 2 s, 4 s, 8 s, 16 s, 32 s, 60 s, or more. – Base network management and publish data communications should not exceed 30% of the available communication bandwidth (100 slots/s max). – The network manager takes into account the service requirements. – The final schedule (not counting the gateway) should have 50% free slots (*i.e.*, allocated for retries and to receive packets).

**Table 3:** Neighbor table parameters [49].

**Content**	**Description**
NeighborUniqueId	Long address of the neighbor device
NeighborNickname	Short address of the neighbor device
Join priority	Depends on the data link layer PDUcontent
TimesourceFlag	Flag indication
Status	Status information regarding this neighbor
BOExp	Backoff exponent for shared link
BOCntr	Backoff countdown for shared link
LastTimeCommunicated	Last time communicated with this neighbor
TimePathFailureTimer	Cyclical path failure timer
AvgRSL	Average received signal level for packets received from neighbor
PacketsTransmited	Number of packets transmitted to the neighbor
MissedAckPackets	Number of packets for which an expected ACK was not received
PacketsReceived	Number of packets received from the neighbor
BroadcastReceived	Number of broadcast packets received from the neighbor

**Table 4:** Comparison of the routing algorithms for WirelessHART.

**Items/Algorithms**	**Han Routing Alg.[52]**	**JRMLR [62]**	**Re-Add [64]**
Objective	Maximize network lifetime	Maximize network lifetime	Robustness and maximize network lifetime
Metric	Average number of hops (degree)	Node data load, transmission energy, residual energy	Quality of the link, residual and transmission energy, degree
Implement uplink, downlink and broadcast graphs	Yes	Builds uplink and states that the downlink would come from setting the algorithm objective to the destination device	Uplink only
Uses node history	No	No	Yes
Implementation and validation	Presents pseudo code for the algorithm; simulation was implemented in [55]	No algorithm is presented, rather, MATLAB® simulation results	No pseudocode is presented; presents simulation results, but no further details on the platform used were given

**Table 5:** Superframe division for the scheduling algorithm presented in [52].

**Link Type**	**First Possible Slot**
Uplink/exclusive slot	0
Uplink/shared slot	$\frac{l_{i}}{4}$
Downlink/exclusive slot	$\frac{l_{i}}{2}$
Downlink/shared slot	$\frac{3*l_{i}}{4}$

**Table 6:** Comparison of WirelessHART scheduling proposals. C-LLF, conflict-aware least laxity first; COOJA, Contiki OS Java.

**Characteristic/ Proposal**	**Dang Alg. [78]**	**Zhang Alg. [79]**	**C-LLF [81]**	**Han Alg. [52]**	**Zhang Policy [82]**
Objective	Accommodate all Txs	Latency and High reliability	Find schedule if there is	Reliability	Time and channel usage
Metric	Publish rate	Degree	Time window (Laxity)	Publish rate	Deadline and Hop count
Multiple superframe	Yes	No	No	Yes	No
Redundancy	Yes	Yes	No	Yes, uses shared slots for possible retries	No
Implementation	No algorithm; uses validation by analytical comparison	Yes, testbed, but no further details on implementation	Yes, optimal and heuristic algorithms and validation with testbeds and simulation	Yes, with simulations in [52] and a full WirelessHART simulation in [55]	No explicit algorithm is presented, but uses MATLAB® and COOJA simulations for validation
Flow differentiation	No	No	No	Yes	No

**Table 7:** Available WirelessHART simulation modules. NS, Network Simulator; OTCL, Object-oriented Tool Command Language.

**Simulator**	**Open Source**	**Language**	**Module Ref.**	**Simulator Ref.**
COOJA	Yes	Java	[102]	[112]
NS-2	Yes	OTCL C++	[55]	[113]
NS-3	Yes	C++ Python	[100,105,114]	[103]
MATLAB® (TrueTime)	No	Simulink blocks	[106,107]	[115]
OPNET	No	OPNET block language	[109]	[111]
